# Frequency of Maxillary Sinus Mucous Retention Cysts in a Central Brazilian Population

**Published:** 2015-09

**Authors:** Evanice Menezes Marçal Vieira, Sylvania de Morais, Carlo Ralph de Musis, Álvaro Henrique Borges, Vinícius Canavarros Palma, Laiane da Silva Basilio, Orlando Aguirre Guedes

**Affiliations:** 1 Dept. of Oral Diagnosis, School of Dentistry, University of Cuiabá, Cuiabá, Mato Grosso, Brazil.; 2 Dept. of Endodontics, School of Dentistry, University of Cuiabá, Cuiabá, Mato Grosso, Brazil.; 3 Dept. of Oral Surgery, School of Dentistry, University of Cuiabá, Cuiabá, Mato Grosso, Brazil.

**Keywords:** Mucous retention cysts, Maxillary sinus, Digital panoramic radiographs, Climate

## Abstract

**Statement of the Problem:**

Mucous retention cysts (MRCs) of the maxillary sinus are lesions with undefined pathogenesis. In recent researches, geographical and climatic aspects have been related as risk factors.

**Purpose:**

The purpose of this study was to determine the frequency of MRCs of the maxillary sinus using panoramic radiographs.

**Materials and Method:**

A total of 631 panoramic radiographs were selected from a secondary database from a private radiology clinic and analyzed by two specialists in dental radiology according to gender, age, month, relative air humidity, and mean temperature.

**Results:**

A total of 87 (6.89%) radiographic images were suggestive of MRCs. Thirty-five MRCs (40.22%) were detected on the right side, 10 (11.49%) on the left side and 42 (48.29%) on both sides. A high frequency was detected in female participants (n=45; 51, 72%), those aged 18-35 years (n=31; 35, 63%) and those from August (n=24; 27.59%) and July (n=22; 25.29%).

**Conclusion:**

The frequency of MRCs was low, and no statistically significant correlation was found between the prevalence of MRCs and the studied variables with the exception of the mean temperature.

## Introduction

Maxillary sinuses, also known as paranasal sinuses, are described as pneumatic (air-filled) cavities.[1] In humans; they are restricted to the skull and can host cystic, inflammatory or neoplastic lesions.[2]

Mucous retention cysts (MRCs) of the maxillary sinus are asymptomatic lesions that are characterized by retention of mucus from the mucous glands of the epithelial lining of the maxillary sinus.[3-4] MRCs are the most frequent lesion of the maxillary sinus[2, 4-7] and affect patients of all races, both genders and various age groups.[3-4,7-10] The pathogenesis of MRCs is undefined,[3, 9-10] although it is strongly related to allergic, inflammatory and infectious processes, trauma, relative humidity and room temperature.[3-4,8-12] Radiographically, MRCs appear as rounded, radiopaque, well-defined images varying in size and located within the maxillary sinus.[3, 8] MRCs may be unilateral or bilateral,[12-13] and they commonly emanate from the sinus floor.[2]

MRCs have been studied in different populations[3, 7-8,11, 14-15] using different methodologies, including analysis during surgical procedures,[13] panoramic radiographs,[3, 9, 12] computed tomography (CT)[5, 10] and cone beam computed tomography (CBCT).[12, 16] Although radiographic images are a two-dimensional representation of a three-dimensional structure, they are the primary resource available for study and represent a non-invasive method for diagnosis and treatment planning for the main procedures relating to the maxilla.[6, 12, 17] Panoramic radiograph has been shown to be a suitable tool for epidemiological studies, and its imaging technique makes it is extremely well suited for imaging findings in the floor or posterior wall of the maxillary sinus.[4, 18]

There is limited information on the occurrence of maxillary sinus abnormalities in the Brazilian population.[7, 12, 16] The geographical and climatic specificities and differences justify the analysis of the frequency of MRCs of the maxillary sinus in the population of Central Brazil, which was performed using panoramic radiographs.

## Material and Method

The panoramic radiographs of 631 patients (1262 maxillary sinuses), 346 female and 285 male, with a mean age 34.65 years, obtained between January and December 2010 were consecutively selected from a private radiology clinic's secondary database (CROIF - Buccal and Maxillofacial Diagnostic Center, Cuiabá / MT) (latitude 15^o^35'56"S, longitude 56^o^ 06'05''W). Patients were referred to the radiology service for different diagnostic reasons. Patients with examinations in which it was not possible to see the maxillary sinus were excluded from the study. This study was approved by the Research Ethics Committee of the University of Cuiabá.

All panoramic images were obtained using the Cranex^® ^equipment direct digital D system (Orion Corp.; Soredex, Helsinki, Finland) and were acquired using a high-frequency generator (40 kHz) sensitized in a CCD sensor measuring 147.5 x 6.1mm with a 96 μm/pixel resolution, a tube voltage of 57 - 85 kV and a current of 10 mA, with 17.6 seconds of exposure time.

The images were analyzed using the panoramic X-ray machine’s own program (DIGORA® for Windows 2.7 software; Orion Corp., Soredex, Helsinki, Finland) on a computer running Microsoft Windows XP Professional operating system SP-2 (Microsoft Corp.; Redmond, WA, USA) with an Intel^®^ Processor Core TM 2 Duo 1.86 Ghz-6300 (Intel Corporation, USA), a glass plate NVIDIA GeForce 6200 TurboCache (NVIDIA Corporation, USA) and EIZO monitor - FlexScan S2000, resolution of 1600 x 1200 pixels (EIZO Nanao Corporation Hakusan, Japan). This analysis was performed by two specialists in dental radiology who were both qualified and experienced in the analysis of panoramic examinations. When consensus was not reached, a third examiner, also a radiologist, made the final decision. The program's brightness and contrast settings were used to optimize the images.

The detection criterion for a maxillary sinus MRC was the detection of a radiopaque image with a dome shape on the floor or other walls of the maxillary sinus.[19] The frequency of MRCs was analyzed by taking into account age, gender, temperature and humidity. All data for relative air humidity and environmental temperature were supplied by INMET (National Institute of Meteorology, Brasília, DF, Brazil) and reported as the mean for each month. The data were organized into frequency tables, and a descriptive analysis was performed with contingency tables and Kendall correlations (5% significance level with 1000 resampled by Monte Carlo simulation).

## Results

**Figure 1:**
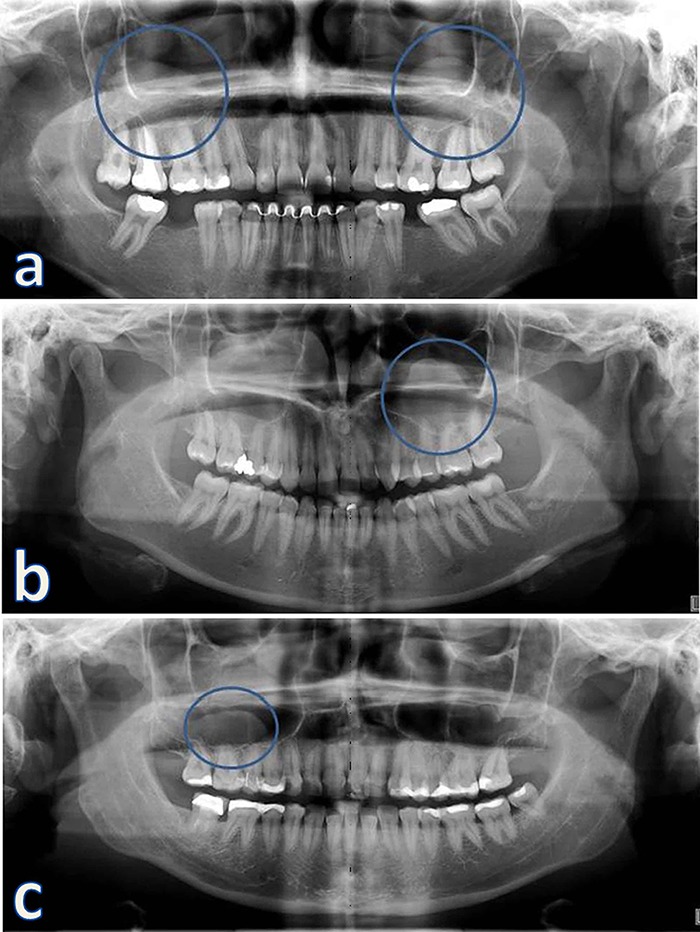
Panoramic radiographs; with images suggestive of a: bilateral mucous retention cyst (circles); b: unilateral mucous retention cyst of the left maxillary sinus and c: unilateral mucous retention cyst of the right maxillary sinus.

A total of 87 (6.89%) radiographic images of the maxillary sinuses of 66 participants were suggestive of MRCs. Of these, 35 (40.22%) were observed on the right side, 10 (11.49%) on the left side and 42 (48.29%) bilaterally. Table 1 shows the distribution of MRCs as a function of gender, age and affected side. Table 2 presents the correlation of MRCs with the month of the year, relative air humidity and temperature. The results of the Monte Carlo test showed that there was a significant correlation only between the frequency of MRCs and the mean temperature. Figure 1 shows examples of panoramic radiographs of patients with unilateral and bilateral MRCs.

**Table 1:** Distribution of mucous retention cysts in relation to gender, age group, and affected side.

	**Right side**		**Left side**	
	**Present n (%)**	**Absent n (%)**	**Present n (%)**	**Absent n (%)**
Gender				
Female	29 (4.60%)	317 (50.24%)	16 (2.54%)	330 (52.30%)
Male	27 (4.28%)	258 (40.89%)	15 (2.38%)	270 (42.79%)
Total	56 (8.88%)	575 (91.13%)	31 (4.92%)	600 (95.09%)
Age group				
≤ 17	9 (1.43%)	120 (19.02%)	5 (0.79%)	124 (19.65%)
18 - 35	21 (3.33%)	173 (27.42%)	10 (1.58%)	184 (29.16%)
36 – 52	16 (2.54%)	188 (29.79%)	11 (1.74%)	193 (30.59%)
≥ 53	10 (1.58%)	94 (14.90%)	5 (0.79%)	99 (15.69%)
Total	56 (8.88%)	575 (91.13%)	31 (4.92%)	600 (95.09%)

**Table 2:** Data of mucous retention cysts with month, mean temperature and mean relative air humidity from January to December 2010 in Cuiabá city (Brazil).

**Month**	**Panoramic (n)**	**MRC (n) (%)**	** Mean temperature (^o^C) **	**Mean humidity (%)**
January	9	4 (4.60%)	30.30	84.50
February	4	1 (1.15%)	28.60	75.00
April	141	16 (18.39%)	28.18	76.29
May	67	8 (9.20%)	25.97	79.26
July	96	22 (25.29%)	16.95	77.54
August	155	24 (27.59%)	21.93	59.17
November	1	0 (0.00%)	27.18	78.50
December	158	12 (13.79%)	29.36	73.33

p-value of Kendall correlation with prevalence = 0.046 for mean temperature and 0.108 for mean humidity; n=631 panoramic radiographs and 87 MRCs (mean 6.89%).

## Discussion

MRCs are defined as mucoid-filled cysts that develop when the seromucous glands of the mucosa of the maxillary sinus become obstructed.[3] MRCs are usually asymptomatic, seldom require treatment and are diagnosed with routine radiographic examinations.[3-4,12] Clinically, they may become significant when they promote the obstruction of the maxillary sinus outflow tract, when they occur with symptoms or when the diagnosis is in doubt.[5] An imaging evaluation of the maxillary sinus, associated with a clinical exam (clinical context), is mandatory in structuring a differential diagnosis and in the establishment of a proper therapeutic protocol.[6, 16, 12] MRC have to be differentiated from several types of sinus diseases, such as sinusitis, mucocele, polyps, benign and malignant neoplasm, and odontogenic cyst.[3, 5-6,12] The polyps are less common lesion, which have been associated with allergic process. Furthermore, polyps are associated with mucous thickening and are multiple lesions while MRC are solitary ones.[17-18] Malignant tumors usually are asymmetrical or have irregular contours. These lesions promote the destruction of the sinus wall, mainly the posterior, and are accompanied by paresthesia.[8-9,19] One finding that is some help in distinguishing odontogenic cyst from MRCs is the lack of a delineating radiopaque border in the latter because it originates within soft tissues outside the maxillary bone.[19]

The motivation for this study was the lack of data on the occurrence of maxillary sinus abnormalities in several regions of Brazil. The study aimed to collect informations about various factors relating to the frequency of MRCs in the city of Cuiabá, a town in the Midwest region of Brazil. The standardization of participants with regard to their medical and dental histories, racial characteristics, facial biotype, environmental influences, dietary habits, and socioeconomic factors could not be established in the present study.

Panoramic radiography was selected because it is a routine exam requested by the dentist that offers the possibility of extensive visualization of the structures of the maxillofacial complex.[10] In addition, panoramic radiographs are a low-cost procedure and are easy to interpret.[20] However, caution is recommended when analyzing and performing absolute measurements and relative comparisons for this type of examination because of the possible overlapping of anatomical structures and the occurrence of false-positive results, distortions, and magnifications.[3, 10, 12, 18, 20] The interpretation of imaging exams demands a familiarity with the anatomical structures of the area under analysis. It is important to be aware of the possible pathologies and anatomical variations and abnormalities that can affect the area.[16] In the present study, two specialists in dental radiology with expertise in the analysis of panoramic radiographs analyzed the images.

The results of the present study revealed a low frequency (6.89%) of MRCs in a Central Brazilian population. This figure is consistent with previous panoramic radiographic studies reported in the literature,[3-4,7-11] which observed a frequency of MRCs ranging from 1.6 to 26.78%. The wide variation in the frequency of MRCS observed in studies conducted in different locations can be explained by the variations in the diagnostic and image interpretation criteria, geographical influences and different population samples.[2, 16]

Regarding gender, MRCs were slightly more frequently found in females than in males (1.08:1). This finding contrasts with the data reported by other authors,[7-9,11, 19] who identified a higher frequency of MRCs among the male population. However, Bhattacharyya[5] observed a female-male ratio of 2.4:1 when determining the incidence of MRCs in a population undergoing evaluation for chronic rhinosinusitis. This study was conducted in a private radiology clinic, and the panoramic exams were undertaken for different diagnoses purposes, such as surgical planning, oral disease diagnosis, and orthodontic diagnosis. The higher frequency of MRCs among women observed in this analysis may be associated with more women than men being referred to the diagnostic imaging service and may not be a true predilection.

In the present study, the age of the patients with radiographic images indicating MRCs ranged from 3 to 88 years, and a high number of MRCs was observed in participants in the 18-35 year age group (31/87). Although several studies suggest that MRCs are found in the greatest proportion in second and third decades of life,[3, 7-9] there have been reports of the highest frequency found beginning in the fourth and even sixth decade of life.[10, 18] Giotakis and Weber[2] performed a comprehensive review of the literature regarding cysts of the maxillary sinus and stated that there is no increased incidence of mucosal cysts for any age group. In the present study, no association between the presence of MRCs and the participants’ age was observed (*p*> 0.05).

For some authors, the frequency of MRCs may vary according to environmental factors, such as the seasons of the year, the mean temperature and the relative air humidity.[3, 10, 21-22] Ruprecht *et al.*[3] investigated the incidence of MRCs in Riyahad (Saudi Arabia) and observed a large number of MRCs in the summer. During this period, high temperatures and a considerable increase in the relative humidity were observed. Martinéz-González *et al.*[10] noted that winter may play a role due to the low temperature and that summer may have an effect due to the use of air conditioners. Carter *et al.*[21] observed a marked increase in prevalence of pseudocysts in Western New York area (EUA) during cold winter months. For the authors, these findings support the concept that seasonal variation may be related to an increased incidence of upper respiratory tract viremias or irritation from dry forced air heating. Casamassimo and Lilly[22] studied the nature of occurrence of MRCs in Iowa (EUA) and observed that the highest frequency of cyst occurred during the period (late winter) often associated with allergic rhinitis and sinusitis.However, after the analysis of 6293 panoramic radiographs in Brasilia (Brazil), Donizeth-Rodrigues *et al.*[12] observed no correlation between the frequency of MRCs and the relative air humidity, mean temperature or month.

In the present analysis, the frequency of MRCs during the months of the year proved to be disproportionate, with numerous cases being observed in August (n = 24, 27.59%) and July (n = 22, 25.29%). February was the month with the lowest frequency (n = 1; 1.15%). Interestingly, in August, the lowest values for the relative humidity and average temperature were recorded. However, the statistical results revealed the presence of a correlation only between MRCs and the mean temperature. It is common, during the winter of Cuiabá (July to September), the phenomenon of temperature inversion. During severe inversions, smog can cover entire metropolitan area of the city and can cause respiratory problems for the inhabitants of those areas. Pollution and variations in relative air humidity can promote an inflammatory response in the respiratory tract, which can cause increased production of acidity, viscosity and consistency of the mucus produced by the airways, leading consequently to a decrease response and / or efficacy of the mucociliary system.[23]

It is important to note that Brazil is a tropical country, and in most regions, there are no drastic temperature changes during the year. In this climate, the four seasons are not well defined, and therefore, comparisons between studies conducted in different geographical areas of Brazil and between studies conducted in other countries should be made with caution. Investigations using other imaging methods, such as cone beam computed tomography, with a greater number of participants should be performed to establish three-dimensional correlations. 

## Conclusion

The frequency of MRCs of the maxillary sinus was low (6.89%). Females and those aged 18-35 years were the groups with the greatest numbers of cases. The peak frequency was in August, and the lowest was in February. Statistical analyses revealed a correlation between the frequency of MRCs and the mean temperature. 
